# Comparison of remifentanil concentrations with and without dexmedetomidine for the prevention of emergence cough after nasal surgery: a randomized double-blinded trial

**DOI:** 10.1186/s12871-021-01358-x

**Published:** 2021-05-04

**Authors:** Ha Yeon Kim, Hyun Jeong Kwak, Dongchul Lee, Ji Hyea Lee, Sang Kee Min, Jong Yeop Kim

**Affiliations:** 1 Department of Anesthesiology and Pain Medicine, Ajou University School of Medicine, 164, World cup-ro, Yeongtong-gu, Suwon, 16499 South Korea; 2 Department of Anesthesiology and Pain Medicine, Gachon University, Gil Medical Center, Incheon, South Korea

**Keywords:** Cough, Dexmedetomidine, Emergence, Remifentanil

## Abstract

**Background:**

Preventing emergence cough after nasal surgery is critical. Emergence cough can provoke immediate postoperative bleeding, which leads to upper airway obstruction. In the present study, we compared the effect-site concentration (Ce) of remifentanil to prevent emergence cough after propofol anesthesia for nasal surgery when remifentanil was or was not combined with dexmedetomidine.

**Methods:**

Forty-seven patients with propofol-remifentanil anesthesia for nasal surgery were randomly assigned to a dexmedetomidine group (Group D, *n* = 23) or a saline group (Group S, *n* = 24). Group D and Group S were infused with dexmedetomidine (0.5 μg/kg) and saline, respectively, for 10 min before the completion of surgery. A predetermined Ce of remifentanil was infused until extubation. Remifentanil Ce to prevent cough in 50 and 95% of patients (EC_50_ and EC_95_) was estimated using modified Dixon’s up-and-down method and isotonic regression. Hemodynamic and recovery parameters were recorded.

**Results:**

The EC_50_ of remifentanil Ce was significantly lower in Group D than in Group S (2.15 ± 0.40 ng/mL vs. 2.66 ± 0.36 ng/mL, *p* = 0.023). The EC_95_ (95% CI) of remifentanil Ce was also significantly lower in Group D [2.75 (2.67–2.78) ng/mL] than in Group S [3.16 (3.06–3.18) ng/mL]. Emergence and recovery variables did not differ between the two groups.

**Conclusion:**

The remifentanil EC_50_ to prevent cough after propofol-remifentanil anesthesia was significantly lower (approximately 19%) when a combination of remifentanil and 0.5 μg/kg dexmedetomidine was used than when remifentanil infusion alone was used in patients undergoing nasal surgery. Therefore, the Ce of remifentanil may be adjusted to prevent emergence cough when used in combination with dexmedetomidine.

**Trial registration:**

ClinicalTrials.gov **(**NCT03622502, August 9, 2018).

## Background

Emergence cough after general anesthesia leads to serious adverse effects including surgical site bleeding, wound disruption, hemodynamic instability, and increased intracranial and intraocular pressure [1]. The prevention of cough in nasal surgery patients is especially important because cough can provoke immediate postoperative bleeding, which leads to upper airway obstruction [2].

Remifentanil has emerged as a medication for cough prevention after general anesthesia. In prior studies, effective remifentanil effect-site concentrations (Ce) under various conditions have ranged from 1.5 to 2.9 μg/mL [3–6]. Although an increasing dose of remifentanil may effectively prevent cough, this drug also increases the incidences of adverse effects including respiratory depression, nausea and vomiting, or delayed emergence [3, 7]. Thus, to decrease remifentanil’s Ce and its side effects when administered alone, co-administration of other adjuvant drugs may prove useful.

Dexmedetomidine is a strong affinity for the α_2_-adrenoreceptor and reduces the use of sedatives and analgesics, though it has little effect on respiratory depression even when used at maximum concentrations [8]. Combination of dexmedetomidine and a low-dose remifentanil administered prior to the end of surgery is reportedly effective in preventing emergence cough without respiratory depression compared to a low-dose of remifentanil alone [9]. In addition, co-administration of dexmedetomidine (0.5 μg/kg) with low-dose remifentanil was not inferior to a high dose of remifentanil alone for preventing emergence cough [10]. However, the effective remifentanil Ce to prevent emergence cough when administered with a single dose of dexmedetomidine has not been evaluated.

The present study estimated the effective remifentanil Ce to prevent emergence cough in 50 and 95% of patients (EC_50_ and EC_95_) administered remifentanil and dexmedetomidine (0.5 μg/kg) and remifentanil alone after propofol anesthesia for nasal surgery.

## Methods

The present prospective trial was conducted with the approval of our Institutional Review Board (AJIRB-MED-OBS-18-170) and registered at ClinicalTrials.gov (ref no.: NCT03622502). After obtaining written informed consent from all participants, patients with American Society of Anesthesiologists physical status I or II, aged between 19 and 65 years, who had planned septoplasty or endoscopic sinus surgeries were enrolled. Participant exclusion criteria were a potentially difficult airway (Mallampati class 3 or 4), use of angiotensin converting enzyme-inhibitors, obesity with body mass index > 35 kg/m^2^, current smoker, a recent upper airway infection, asthma, and uncontrolled hypertension. According to a randomization generator (http://www.random.org), patients were randomized to a dexmedetomidine group (Group D) or a saline group (Group S).

When the patient arrived at the operating room, anesthetic monitoring including non-invasive blood pressure (BP) measurement, electrocardiography, and peripheral pulse oximetry was started. The anesthetic depth was assessed by attaching a bispectral index (BIS) sensor to the participant’s forehead. For anesthesia induction, target-controlled infusion was started (Ce of propofol at 5.0 μg/mL and Ce of remifentanil Ce at 4.0 ng/mL) using an infusion device (Orchestra, Fresenius Vial, France). Two minutes after rocuronium (0.6 mg/kg) injection, endotracheal intubation was performed using a 7.0 mm and 7.5 mm cuffed tube in men and women, respectively, with a cuff pressure of 20–25 mmHg.

Anesthesia was maintained with Ce of propofol at 2.0–3.0 μg/mL and Ce of remifentanil at 3.0–5.0 ng/mL. Anesthetic depth was adjusted from a BIS value of 40 to 60. Fluctuations of intraoperative heart rate (HR) and BP were adjusted to within 20% of the baseline (before the induction of anesthesia). When HR dropped below 45 bpm, atropine (0.5 mg) was administered. When the mean BP decreased to less than 20% of the baseline, ephedrine (6 mg) was administered.

Dexmedetomidine (0.5 μg/kg) in Group D and the same volume of normal saline in Group S were infused using a syringe pump for over 10 min, before completion of surgery. Upon completion of surgery, propofol infusion was halted. Throughout emergence, remifentanil infusion of predetermined Ce was continued for more than 15 min until extubation. Drugs were administered by one researcher (JY Kim) according to the patient’s group identity (dexmedetomidine or normal saline and a pre-determined Ce of remifentanil). Patients’ degree of muscle relaxation was estimated using train-of-four (TOF) monitoring. When the TOF ratio was more than 90%, neostigmine (0.02 mg/kg) and glycopyrrolate (0.004 mg/kg) were injected. Subsequently, assisted ventilation with 100% of inspired oxygen was initiated in response to spontaneous patient breathing. When the patient showed spontaneous eyes opening or response to a verbal command, we confirmed that their spontaneous breathing was sufficient and removed their endotracheal tube. Thereafter, remifentanil was stopped and a facial mask delivering 100% oxygen was applied. The patient was moved to a post-anesthetic care unit (PACU) after confirming the adequacy of their consciousness and respiration over a 5-min period. In the PACU, the patient was assessed for postoperative nausea and vomiting (PONV). Postoperative pain was quantified using a numeric rating scale (NRS), ranging from 0 to 10 (0 = no pain, 10 = worst possible pain). If the patient suffered from pain rated worse than a 5 or requested painkiller administration, fentanyl (50 μg) was injected. Sedation was also evaluated using a modified Wilson sedation scale [11]. When the modified Aldrete score was ≥9, patients were moved to the ward [12].

Patients were sequentially enrolled using a Dixon’s up-and-down allocation approach, as previously [13]. Patient enrollment continued until both groups reached at least 20 patients and six success-failure pairs. Cough was defined as a sudden expulsion of air with abdominal muscle contraction and classified into one of the four grades (grade 0 = no cough, grade 1 = single cough, grade 2 = more than one episode of non-sustained cough, grade 3 = sustained and repetitive cough). Cough was assessed between the end of surgery and 5 min after extubation. The Ce of remifentanil was initiated with 2.0 ng/mL in each group. The Ce of remifentanil of the next patient was determined by the presence of coughing in the previous patient. If the patient had no cough or a single cough (grade 0 or 1), we defined this as successful prevention of cough, and the pre-determined Ce of remifentanil for the next patient was lowered by 0.4 ng/mL. If cough was not prevented successfully (grade 2 or 3), we determined the result to be a failure at preventing cough, and the pre-determined Ce of remifentanil for the next patient was increased by 0.4 ng/mL.

During the operations, data on the Ce for propofol and remifentanil, mean BP, HR, pulse oximetry saturation (SpO_2_), BIS value, respiratory rate, and end-tidal CO_2_ (EtCO_2_) were collected at seven time points, namely, baseline (T0), immediately before (T1) and after (T2) the start of dexmedetomidine or saline infusion, upon operation completion (T3), at eye opening (T4), and immediately (T5) and 5 min (T6) after extubation. The intraoperative use of medications to control BP or HR was also recorded.

Cough was assessed by one researcher (HY Kim) who was blinded to patients’ group allocations and the predetermined Ce of remifentanil. The times elapsed between stopping propofol administration to eye opening (time to eye opening) and from stopping propofol administration to extubation (time to extubation) were recorded. For 5 min after extubation, hypoventilation (respiratory rate < 8 breaths/min), laryngospasm, and desaturation (SpO_2_ < 95%) were recorded. In the PACU, respiratory rate, PONV, pain scores using the NRS, Aldrete scores, sedation scale scores, and stay duration were recorded.

### Statistical analyses

The EC_50_ and EC_95_ of remifentanil to prevent cough in Group D were the primary study outcomes. To obtain the EC_50_ by Dixon’s method, minimum six success-failure pairs and 20 patients were needed [14]. The EC_50_ of remifentanil was obtained by the mean value of the mid-point for each failure-to-success pair. In a previous study on nasal surgery using Dixon’s method, the standard deviation (SD) of EC_50_ of remifentanil Ce to prevent emergence cough was 0.38 ng/mL [4]. Since the step size of Ce should be larger than the previous SD, we set the change of the adjacent dose of remifentanil to 0.4 ng/mL. To obtain the EC_95_ of remifentanil, the isotonic regression method using a pooled-adjacent-violators algorithm and a bootstrapping approach was also used, as previously [15]. No overlap between two EC_95_ values at 95% confidence interval (CI) was considered a significant difference [16].

Categorical variables were analyzed using the chi-squared or Fisher’s exact tests and presented as numbers (frequency). Continuous variables were analyzed using independent t-tests or Mann-Whitney U tests and presented as means ± SDs or medians (25th to 75th quartile). Measured variables were repeatedly analyzed using the linear mixed model. When the model revealed a significant interaction between group and time, a post-hoc analysis was performed to identify time points which differed significantly. The variables were considered statistically significant when the *P*- value was less than 0.05. Statistics were analyzed with SPSS (version 25.0, IBM Corporation, Armonk, NY, USA) and R (version 3.2.5).

## Results

**Fig. 1:**
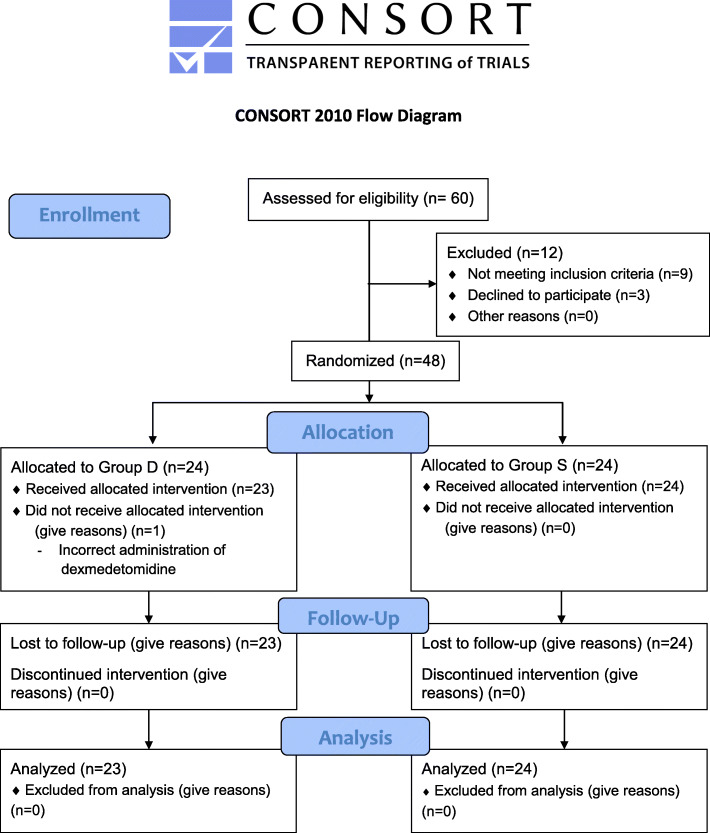
The CONSORT flow diagram

Forty-eight patients were enrolled between August 2018 and March 2019. One patient was withdrawn due to incorrect initiation of dexmedetomidine. Twenty-three patients in Group D and 24 patients in Group S included in this study (Fig. 1). Preoperative patients’ characteristics and intraoperative details were comparable between the two groups (Table 1).

**Table 1:** Preoperative and intraoperative patients’ characteristics

	Group D (*n* = 23)	Group S (*n* = 24)	*P*-value
Sex, male n (%)	14 (61)	19 (79)	0.636
Age, years	40 ± 12	40 ± 14	0.875
Weight, kg	74 (66–84)	73 (64–76)	0.442
Height, cm	170 (160–176)	172 (166–182)	0.248
ASA classification (I/II), n	19/4	18/6	0.724
Surgery time, min	35 (25–40)	30 (25–44)	0.765
Anesthesia time, min	70 (60–75)	70 (60–89)	0.579

Values are mean ± standard deviation, median (25th – 75th quartile), or number (%)

*ASA* American Society of anesthesiologist

**Fig. 2:**
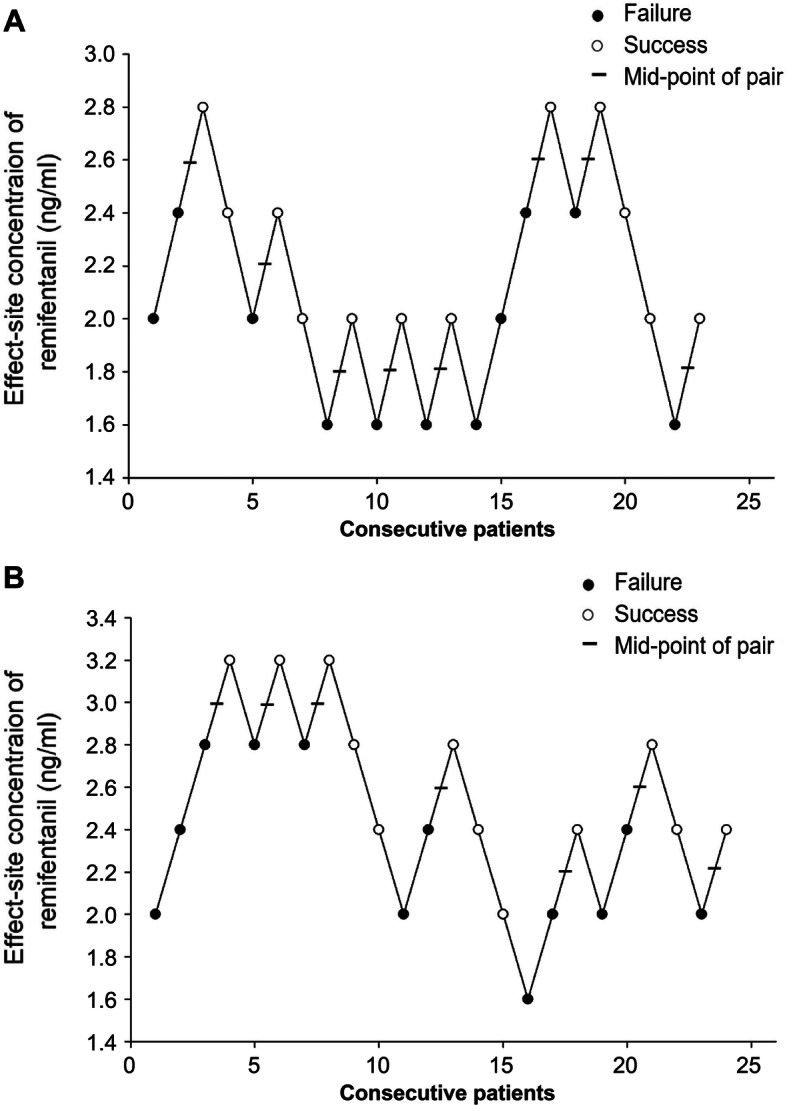
Effect-site concentration of remifentanil by Dixon’s method in Group D (**a**) and in Group S (**b**)

Success and failure rates to prevent emergence cough in consecutive patients are presented in Fig. 2. EC_50_s were calculated by the Dixon’s method from eight failure-success pairs in Group D and from seven failure-success pairs in Group S. The EC_50_ for remifentanil was significantly lower in Group D than in Group S (2.15 ± 0.04 vs. 2.66 ± 0.36 ng/mL, respectively, *P* = 0.023). The EC_95_ (95% CI) for remifentanil was also significantly lower in Group D than in Group S [2.75 (2.67–2.78) vs. 3.16 (3.06–3.18) ng/mL, respectively], and their 95% CIs did not overlap.

During surgery, repeated measure variables including MBP, HR, SpO_2_, BIS value, respiratory rate, and EtCO_2_ had similar trends over time in both groups (all *p* values > 0.05) (data not shown). The number of patients who were administered ephedrine was comparable between the two groups [9 (39.1%) in Group D vs. 11 (45.8%) in Group S, *p* = 0.642]. One patient in Group D received atropine.

During emergence, time to eye opening, time to extubation, and respiration rate were comparable between the two groups. Hypoventilation within 5 min of extubation occurred in six patients in Group D and in nine patients in Group S (*P* = 0.401). This hypoventilation was transient in all patients and recovered with respiratory encouragement (Table 2).

**Table 2:** Emergence and recovery parameters

	Group D (*n* = 23)	Group S (*n* = 24)	*P*-value
During anesthetic emergence			
Time to eye opening, sec	560 (490–670)	565 (453–714)	0.975
Time to extubation, sec	670 (630–750)	690 (540–795)	0.775
Respiration rate, breaths/min			
Immediately after extubation	10 (9–12)	12 (9–13)	0.412
5 min after extubation	12 (12–13)	12 (10–13)	0.360
Hypoventilation, n (%)	6 (26)	9 (38)	0.401
In the post-anesthesia care unit			
Pain score, NRS	2 (2–3)	2 (2–3)	0.644
Rescue analgesics, n (%)	2 (10)	2 (9)	0.456
PONV, n (%)	4 (18)	2 (9)	0.414
Stay duration, min	30 (30–40)	30 (30–40)	0.745

Values are median (25th – 75th quartile), or number (%)

*NRS* Numeric rating scale (0 = none, 10 = the worst), *PONV* Postoperative nausea and vomiting

In the PACU, NRS for postoperative pain, the patients’ number receiving rescue analgesics, PONV, and stay duration were comparable between the two groups. Respiratory rate, Aldrete scores, and sedation scale scores also did not interact significantly with time and group (data not shown).

## Discussion

In this study, we evaluated remifentanil’s Ce to prevent emergence cough with and without co-administration of dexmedetomidine (0.5 μg/kg) after propofol anesthesia. The combined infusion of dexmedetomidine and remifentanil significantly reduced remifentanil EC_50_ and EC_95_ measures by 19 and 13%, respectively, compared to remifentanil infusion alone. In addition, the combined use of these drugs did not delay the time to awakening or extubation and did not aggravate respiratory depression.

Cough is mediated by peripheral nerve terminals within the airway walls and by central vagus afferent nerves in the nodose ganglia or bodies of the jugular [17, 18]. Several antitussive agents are known to inhibit peripheral cough pathways (e.g., local anesthetics), central cough pathways (e.g., gamma-aminobutyric acid agonists), or both cough pathways (e.g., opioids) [17, 18]. Of these, remifentanil is the antitussive agent of choice during surgery due to its uniquely rapid action without accumulation [7]. However, although remifentanil has a dose-dependent antitussive effect, it also has dose-dependent adverse effects such as respiratory depression, nausea and vomiting, muscle rigidity, pruritus, or delayed emergence [3, 7].

In recent years, the application of dexmedetomidine, which has respiratory preserving properties, has grown during anesthesia [19]. Given this, several studies have assessed the effectiveness of dexmedetomidine to prevent cough [20–24]. At present, the results regarding the antitussive effects of dexmedetomidine have been controversial. Several studies have reported that dexmedetomidine may not prevent cough better than remifentanil, midazolam, or even saline [20–22]. However, other studies have reported that dexmedetomidine may prevent cough better than placebo (saline) [23, 24] and that it may have dose-dependent antitussive effects [23]. Pretreated dexmedetomidine 0.6 μg/kg bolus intravenous infusion over 10 min could reduce fentanyl-induced cough effectively without side effects in a previous study [25]. In addition, Lee et al. also found that the addition of a single dose of dexmedetomidine to a low-dose infusion of remifentanil during emergence from sevoflurane-remifentanil anesthesia was effective in attenuating cough after thyroid surgery [9]. Although the criteria for enrolling patients and statistical methods in the Lee et al.’s study are different from those of our study, we also demonstrated that dexmedetomidine combined with remifentanil may be highly effective at preventing cough compared to remifentanil alone after nasal surgery.

According to the manufacturer, dexmedetomidine has a wide range of dosage in a bolus (0.5 to 2.0 μg/kg over 10 min) and infusion (0.1 to 1.5 μg/kg/h) depending on the clinical situation, including general anesthesia, sedation in the intensive care unit, or procedural sedation. Although bradycardia and hypotension may occur with bolus doses [19], a bolus administration of dexmedetomidine is still considered as an attractive method because it is easy and simple. Since Guler’s et al. presented a study that showed that administration of a single dose of dexmedetomidine (0.5 μg/kg) at the end of surgery reduces airway and circulatory reflexes during extubation [24], this administration method has become popular in clinical practices for smooth emergence [9, 10, 23, 26]. Hence, we set the administration dose of dexmedetomidine to 0.5 μg/kg in this study.

The present study revealed differences of 0.4–0.5 ng/mL in remifentanil EC_50_ and EC_95_ between the two groups. In addition, a reduced Ce of remifentanil when dexmedetomidine was combined did not delay emergence time (from eye opening to extubation) compared to the use of remifentanil alone. In this study, the remifentanil EC_95_ after nasal surgery when remifentanil was used alone was 3.16 ng/mL. This remifentanil Ce was a substantially higher dose than that reported previously in the context of thyroid surgery (2.14 ng/mL) or brain tumor surgery (2.51 ng/mL) [27, 28]. Meanwhile, Choi et al. [4] found that the ideal remifentanil EC_95_ to prevent cough after nasal surgery was 2.94 ng/mL, which is comparable to that reported in our study. Choi et al. suggested that coughing was more frequent after nasal surgery than after other types of surgery, potentially because of chronic inflammation in the nasal mucosa, perioperative mechanical irritation, and pharyngolaryngeal stimulation by blood. Thus, the type of surgery could be a factor determining the optimal remifentanil Ce to prevent emergence cough.

Despite our findings, the use of high concentrations of remifentanil (e.g., above 3.0 ng/mL) to prevent cough may not be practical given that remifentanil infusion during emergence under propofol anesthesia may increase the hypnotic effects of propofol and respiratory depression [29]. In a previous study, remifentanil infusion at 3.0 ng/mL during laryngomicroscopic surgery after propofol anesthesia led to a higher incidence of hypoventilation and longer extubation time during emergence than remifentanil infusion at 2.6 ng/mL or less [6]. This result indicates that the combined use of dexmedetomidine and remifentanil for preventing emergence cough is feasible in clinical settings.

In this study, the combined use of remifentanil and dexmedetomidine did not attenuate hemodynamic changes during extubation better than remifentanil alone. This finding is contrary to previous reports in which hemodynamic changes were attenuated better with combined dexmedetomidine (0.5 μg/mL) and remifentanil (1 ng/mL) than with remifentanil infusion alone [9]. The mean Ce for remifentanil in the present study was 2.1 in Group D and 2.5 ng/mL in Group S. Remifentanil attenuated hemodynamic changes during emergence in a dose-dependent manner [5]. Thus, relatively high doses of remifentanil may have offset the cardiovascular effects of dexmedetomidine. Meanwhile, recovery profiles including respiratory rate were not different between the two groups, paralleling findings from a previous study [10]. The previous study suggested that remifentanil plays a major role in regulating respiratory profiles when combined with dexmedetomidine because it does not worsen the respiratory depression induced by remifentanil [10].

There were some limitations in the present study. First, although the Dixon’ up-and-down method allows for good median estimation, it is a simple strategy. Because such median estimations depend on the chosen pairs (e.g., success-failure pairs or failure-success pairs) and clinical circumstances, the EC_50_ is a relative and not absolute value. Second, our sample size was estimated using the Dixon’ up-and-down allocation approach and may be insufficient to confirm differences in secondary outcomes between the two groups. Third, all cases included in this study underwent propofol anesthesia. Since many hypnotic agents even at sub-hypnotic concentrations influence airway reflexes [30], a residual concentration of propofol at extubation can affect cough reflex. Therefore, different results may emerge in cases which utilize inhalational anesthesia. Lastly, we cannot completely rule out the negative recovery parameters and adverse effects resulting from the use of dexmedetomidine during the perioperative period. This study did not show that dexmedetomidine has a negative effect on the recovery of anesthesia. However, dexmedetomidine (0.5 μg/kg) combined with remifentanil 10 min before the end of surgery prolonged the time of extubation in an earlier study [9]. Because the included patients in this study were approximately 40 years old, this difference might be due to the age difference of included patients. Therefore, our results cannot be extrapolated to the elderly. In addition, when dexmedetomidine was co-administered with other sedatives or analgesics, the sedative or hemodynamic effects could be more pronounced [19]. Considering that dexmedetomidine has a long metabolic time, co-administration of dexmedetomidine and remifentanil should be used with caution in the elderly and co-morbid patients.

## Conclusions

The Ce for remifentanil to prevent emergence cough after propofol anesthesia for nasal surgery was significantly lower when a single dose of dexmedetomidine (0.5 μg/kg) was co-infused with remifentanil than when remifentanil was administered alone. The Ce of remifentanil may be adjusted to prevent emergence cough when used in combination with dexmedetomidine.

## Data Availability

The datasets generated and analyzed during the present study are available from the corresponding author on reasonable request.

## Abbreviations

BIS: Bispectral index
BP: Blood pressure
Ce: Effect-site concentration
CI: Confidence interval
EC_50_: Effective concentration in 50% of patients
EC_95_: Effective concentration in 95% of patients
ETCO_2_: End-tidal CO2
HR: Heart rate
NRS: Numeric rating scale
PACU: Post-anesthesia care unit
PONV: Postoperative nausea and vomiting
SD: Standard deviations
SPO_2_: Pulse oximetry saturation
TOF: Train-of-four
